# Quantitative Assessment and Regulation of Passage and Entrance Attraction Efficiency of Vertical-Slot Fishway on Heishuihe River in Southwest China

**DOI:** 10.3390/ani14162365

**Published:** 2024-08-15

**Authors:** Jiawei Xu, Dongqing Li, Xiaozhang Hu, Yilin Jiao, Jianping Wang, Yujiao Wu, Chenyu Lin, Senfan Ke, Tianxiang Bai, Nannan Wang, Bingjun Liu, Xiaotao Shi

**Affiliations:** 1 School of Civil Engineering, Sun Yat-sen University, Guangzhou 510275, China; xujiawei928@163.com; 2 Pearl River Water Resources Research Institute, Pearl River Water Resources Commission, Guangzhou 510611, China; hbhuxz@163.com (X.H.); wangm13@126.com (J.W.); 3 Hubei International Science and Technology Cooperation Base of Fish Passage, China Three Gorges University, Yichang 443002, China; lidongqingya@163.com (D.L.); 13669078519@163.com (Y.W.); linchenyu@ctgu.edu.cn (C.L.); 201708150011005@ctgu.edu.cn (S.K.); 202107130021015@ctgu.edu.cn (N.W.); 4 State Key Laboratory of Hydraulics and Mountain River Engineering, Sichuan University, Chengdu 610065, China; jiaoyilin@stu.scu.edu.cn; 5 China Three Gorges Renewables (Group) Co., Ltd., Gansu Branch, Lanzhou 730070, China; 18298401328@163.com

**Keywords:** vertical-slot fishway, quantitative assessment, fishway entrance, fish passage efficiency, environmental factors

## Abstract

**Simple Summary:**

Fish passage facilities are crucial for restoring river connectivity and protecting ecosystems. This study quantitatively assessed the upstream migration of fish under various environmental conditions. In the Heishuihe River downstream of the Songxin Dam, 19 fish species were monitored, with 15 species reaching the fishway entrance and 12 successfully passing through. The entrance attraction and passage rates of the vertical-slot fishway at Songxin were 15.7% and 40.42%, respectively. The results indicate that May is the optimal period for fish migration, with better performance observed during nighttime than daytime. Optimal entrance attraction occurred at a flow rate of 6–7 m^3^/s and a temperature of 19–20 °C, while the best passage efficiency was achieved at a flow rate of 0–0.5 m^3^/s and a temperature of 17–20 °C. A multifactorial Cox regression model identified diurnal rhythms, release location, temperature, and flow rate as primary hindering factors, while body length and water level were found to be promoting factors. A nomogram was developed to predict the success rates of fishway attraction and passage based on these key factors. This study provides theoretical and data support for optimizing fishway operations and offers scientific insights into ecological restoration.

**Abstract:**

Fish passage facilities are essential for restoring river connectivity and protecting ecosystems, effectively balancing economic and ecological benefits. Systematic and comprehensive monitoring, assessment, and optimized management are therefore crucial. This study quantitatively evaluated the entire upstream migration process of fish from the downstream river to the entrance and exit of the fishway and investigated the upstream movement patterns of fish under various environmental factors. A total of 19 fish species were monitored in the Heishuihe River downstream of the dam, with 15 species reaching the fishway entrance and 12 species successfully passing through it. The entrance attraction and passage rates of the vertical-slot fishway at the Songxin hydropower station were 15.7% and 40.42%, respectively. The best upstream performance was observed in May, with fish demonstrating better upstream timing and speed during nighttime compared to daytime. Specifically, the highest entrance attraction efficiency was recorded at a flow rate of 6–7 m^3^/s and a temperature of 19–20 °C, while the optimal passage efficiency was observed at a flow rate of 0–0.5 m^3^/s and a temperature of 17–20 °C. Additionally, a multifactorial Cox proportional hazards regression model was constructed to identify key factors influencing the probability of fishway entrance attraction and successful passage. The model elucidated the impact patterns of these key factors on fish upstream migration, ultimately generating an alignment diagram for prediction and control. This study provides a theoretical foundation and data support for developing optimized operational schedules for fishways. The findings offer a more comprehensive and systematic approach for monitoring and evaluating fish passage facilities, serving as a scientific basis for ecological restoration and fish conservation in this region and similar areas.

## 1. Introduction

River connectivity and ecosystem integrity play a crucial role globally in maintaining biodiversity, supporting sustainable development, and addressing climate change. The Jinsha River Basin in southwest China is crucial for water resources and ecosystem supply. However, over the past few decades, extensive hydraulic engineering projects in the region have profoundly impacted the ecological environment. Large-scale reservoir construction, dam building, and flow regulation measures have directly disrupted river ecosystems and severed river connectivity [1,2]. This disturbance has posed substantial challenges in terms of biodiversity, fish migration, and habitat restoration, and has driven some fish species to the brink of extinction [3,4,5]. To mitigate or avoid the adverse effects of hydropower development on river connectivity, researchers and habitat restoration practitioners have made extensive efforts to devise strategies. These strategies include fish stocking, dam removal, wetland restoration, and construction of fish passage facilities to restore the ecological functions of rivers and safeguard valuable fish resources [6,7,8]. Among these strategies, fish passage facilities are widely applied because of their advantages in maintaining dam functions, protecting ecosystems, their broad applicability, and cost-effectiveness [9].

Based on principles such as structural functionality and applicability, engineers and ecologists have categorized fish passage facilities into various types, such as nature-like, Denil, pool-and-weir, vertical-slot, and automatically operated locks and lifts [10,11]. Vertical-slot fishways have particularly become popular and widely used as they are versatile in accommodating different types of dams and weirs and can facilitate successful migration for fish with different life habits [12]. Although increasing attention has been paid to fishway construction and the potential of these fishways to allow fish passage, many fishways continue to lack monitoring and quantification of the fish passage efficiency. Monitoring and assessing fishway effectiveness can help understand the ecological dynamics of migrating fish within dam systems [13,14]. Comprehensive monitoring of the fish passage efficiency can facilitate pool diagnostics, structural optimization, and overall improvement in fish passage effectiveness [15].

In the early stages of fishway construction, the primary focus of engineering researchers is the design, construction, and effectiveness assessment of fishways. According to experts, the successful upstream migration of target species through fishways indicates their effectiveness. Previously applied monitoring methods typically involved the use of videos, acoustics, and radio frequency identification (RFID) to track fish quantity and species at fishway outlets and validate their effectiveness [16,17]. However, these methods did not determine the attraction efficiency at fishway entrances or the fish upstream migration patterns within the fishways. With the deepening understanding of aquatic ecosystems, scholars have recently started emphasizing the significance of the entrance attraction efficiency in fishways and corresponding control measures for optimizing their performance [18]. The entrance attraction efficiency is a crucial influencing factor that determines whether fish can be effectively lured into fishways [19]. This parameter ultimately impacts the overall effectiveness of fish passage. To address this issue, various factors influencing fish behavior and their responses to entrance conditions in fishways must be comprehensively analyzed [9,20]^.^ Therefore, to quantitatively assess fishway effectiveness and fish passage efficiency, systematic monitoring of fishway effectiveness and the establishment of a comprehensive set of metrics are urgently required. This assessment includes examining the entire process from the downstream river section through the fishway entrance to the fishway exit. Such assessments offer a theoretical basis and data support required for developing optimized operational and scheduling plans for fishways.

Fish can be assisted in their successful passage over dams by identifying the factors that drive their upstream behavior [21], which include biological elements (species specificity and fish biology) and environmental factors (flow rates, velocity, water levels, temperature, and diurnal rhythms). These factors typically serve as critical determinants of fish migration [22,23,24]. Through a series of experiments, researchers have recently explored fish behavior under different environmental conditions. For instance, within culvert fishways, brook trout (*Salvelinus fontinalis*) demonstrated a significantly higher rate of upstream attempts under high-flow conditions than under low-flow conditions [25]^.^ Meanwhile, the spotted barbel (*Parazacco spilurus* (Günther, 1868)) preferred to avoid areas with high flow velocities and turbulence during their upstream migration and favored regions with lower flow velocities [26]. Additionally, elevated water temperatures can improve the motivation and success rate of American shad (*Alosa sapidissima*) passing through fishways [27]. Nevertheless, most studies have focused on analyzing fish behavior in response to individual environmental factors, often neglecting the interplay among multiple environmental elements. Consequently, more in-depth research is required to decipher the behavioral patterns of fish under the influence of multiple environmental factors. Such investigations will aid accurate identification of crucial factors affecting the fish upstream migration efficiency. This, in turn, will help in understanding the fundamental reasons behind suboptimal fishway performance more precisely and facilitate the development of effective optimization and control measures. Therefore, identifying key biological and non-biological factors influencing successful fish passage is crucial for the design of optimized fish passage facilities.

Through fish resource surveys, this study focuses on the Songxin vertical-slot fishway in the Heishuihe River, a Jinsha River tributary, to investigate the temporal and spatial distribution patterns of fish resources downstream of the hydroelectric station. We conducted fishway pool diagnostics and assessed fish passage effectiveness, thereby verifying the fishway’s efficacy and quantifying both the entrance attraction and passage efficiencies. For this assessment, monitoring techniques such as collection nets and RFID were employed. Finally, a Cox proportional hazards regression model was constructed to evaluate the fishway’s passage effectiveness, identify critical factors influencing fish passage, and elucidate the fish upstream migration patterns under varying environmental conditions. This study traced the complete upstream passage process of fish from the downstream river to the fishway entrance and exit. A systematic indicator system and model were established for assessing, diagnosing, and predicting the entrance attraction and passage efficiencies of the fishway. The research methods and findings offer a theoretical foundation for river connectivity restoration, reservoir scheduling strategies, and optimized fish passage facility design. Based on a detailed analysis of the fishway’s role in mitigating the impacts of hydraulic engineering on ecosystems, this study provides scientific insights into ecological restoration and fish conservation in this region and similar areas.

## 2. Materials and Methods

### 2.1. Study Area

**Figure 1:**
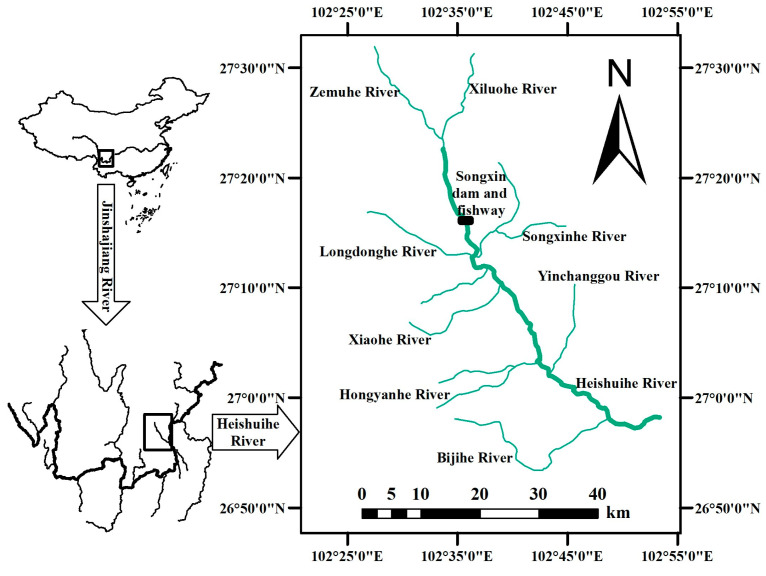
Geographic location of research area.

The Heshuihe River, a primary left-bank tributary of the Jinsha River, is situated within the Sichuan province of southwest China. This river is a priority conservation area for fish habitats within the Baihetan Hydropower Station reservoir. The mainstream of the Blackwater River currently has three hydroelectric stations. This study was conducted at the Songxin Power Station (Figure 1). To facilitate fish migration upstream and downstream of the dam and to restore river connectivity, this power station has built a vertical-slot fishway and a supplementary water diversion channel. The fishway is present on the left bank of the dam and can accommodate a flow rate of 0.176 m^3^/s with a flow velocity of 0.75 m/s. The upstream designed water level is 1067.16 m, while the downstream level is 1063.87 m. The fishway has a slope of 1.80%. The dimensions of the fishway pool are 2 m in length by 1.6 m in width. To enhance the fish attraction efficiency at the fishway entrance, an engineering solution involving the supplementary water diversion channel is implemented, thereby expanding the attraction flow range at the entrance.

### 2.2. Experimental Methods

#### 2.2.1. Fish Resource Survey

In order to analyze the spatiotemporal distribution patterns of fish resources downstream of the Songxin Dam, this study established 14 sampling points along a 2.5 km stretch downstream of the Heshuihe River based on principles such as river morphology, substrate composition, flow velocity ranges, depth variations, water visibility, and temperature fluctuations. Fish resource surveys were conducted during the fish migration period (March to May).

During each sampling event, morphometric measurements (total length, fork length, standard length, and weight) of the captured fish were conducted and recorded. The collected samples were taxonomically identified on the basis of references such as “The Fishes of Sichuan” [28] and “Systematic Synopsis of Chinese Fishes” and consultation with relevant experts. The fish caught during the experiment were released back into the upstream section of the river after data collection to ensure that the released fish would not affect subsequent trials.

#### 2.2.2. Net Collection Experiment

**Figure 2:**
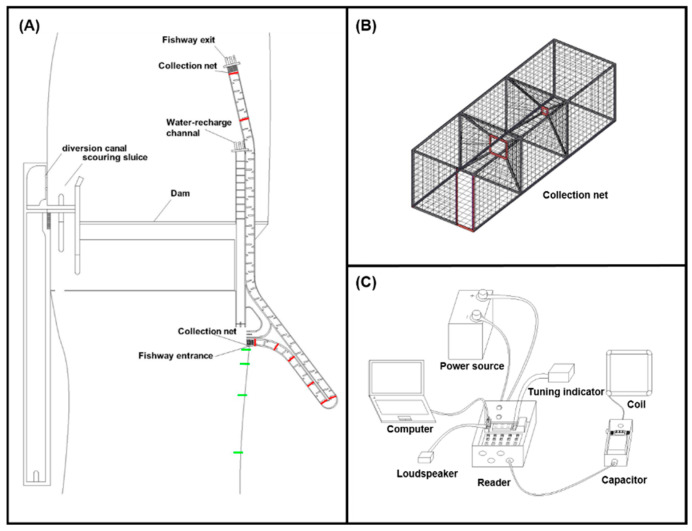
Schematic diagram of vertical slot fishway and monitoring devices in the Heishuihe River. (**A**) Layout of monitoring devices. The green box represents the location of fish release in the river, and the red box represents the arrangement of RFID devices. (**B**) Collection net device schematic. The device has a series of narrowing net openings and a constricted cross-section to collect fish. (**C**) Radio frequency identification (RFID) device schematic.

To understand the fish upstream patterns within the dam’s downstream river entrance of the fishway exit of the fishway route, timed net collection experiments were conducted between March and May 2022. These experiments helped in monitoring the types and quantities of fish passing through the fishway entrance and exit (Figure 2) for 6 days each month, with 3 days for monitoring the entrance and 3 days for monitoring the exit. Each monitoring session lasted 24 h. For this experiment, two collection nets were employed: one at the entrance and one at the exit of the vertical-slot fishway pool chambers. To prevent the escape of the captured fish, the collection nets had progressively narrowing mesh structures and narrowed sections. Fish were intercepted and captured on the basis of their natural upstream swimming behavior. Morphometric measurements and taxonomic identification of each capture were performed, and the results were recorded. After data collection, all captured fish were released back into the original river section.

#### 2.2.3. Fish Passage Efficiency Experiment

To determine the effectiveness of the vertical-slot fishway and identify key factors affecting fish upstream migration, sub-adults *Schizothorax wangchiachii* (mean ± SD, length = 242.46 ± 35.07 mm, weight = 242.46 ± 35.07 g), a primary conservation target in the Heshuihe River, were selected. By injecting PIT tags into the fish, half-duplex RFID technology was used for real-time monitoring of fish upstream migration. When a fish passed through or entered the range of the antenna, the system recorded the detection time and the tag number associated with that fish.

The fishway passage efficiency experiment was categorized as river release and fishway release (Figure 2). In the river release experiments, fish were released at 1, 10, 20, and 30 m away from the fishway entrance to monitor their upstream migration behavior when they entered the fishway. The fishway release experiment involved blocking the fishway entrance with a net, capturing the fish, and releasing the captured fish into the first-level pool within the fishway to observe their upstream patterns within different chambers from the entrance to the exit. Both experiments were conducted three times, with the release of 100 tagged fish in each experiment. Each experiment had a 7-day monitoring period, during which water temperature, water depth, flow rate, and other data were continuously recorded.

### 2.3. Data Analysis

To quantitatively assess the fish passage efficiency, the attractability of the fishway entrance was evaluated using indicators such as the fishway attraction rate, attraction speed, and attraction time. Subsequently, the overall fishway and pool passage effectiveness were assessed using metrics such as the fishway passage rate, passage time, and fishway passage speed.

P*_e_* = N_1_/N_0_ × 100% (1)

In the equation, P*_e_* represents the fishway entrance attraction rate, N_0_ stands for the number of fish released in the downstream river section, and N_1_ indicates the number of fish that successfully passed through the antenna at the entrance of the fishway’s first pool chamber.

T*_a_* = T_1_ − T_0_ (2)

In the equation, T*_a_* represents the fishway entrance attraction time, T_0_ stands for the time when fish are released in the downstream river section, and T_1_ indicates the time when fish successfully pass through the antenna at the entrance of the fishway’s first pool chamber.

P*_ij_* = N*_i_*/N*_j_* × 100% (3)

In the equation, P*_ij_* represents the passage rate from sections *j* to *i* in the fishway, and N*_i_* and N*_j_* denote the number of fish that pass through the antenna at sections *i* and *j*, respectively.

T*_ij_* = T*_i_* − T*_j_* (4)

In the equation, T*_ij_* represents the passage time from section *j* to section *i* in the fishway, and T*_i_* and T*_j_* represent the timing of fish passing through antennae *i* and *j*, respectively.

V*_ij_* = (L*_i_* − L*_j_*)/T*_ij_* (5)

In the equation, V*_ij_* represents the passage velocity from sections *j* to *i* in the fishway, and L*_i_* and L*_j_* represent the distances from antennae *i* and *j*, respectively, to the fishway entrance.

To identify key factors influencing the fishway passage efficiency and quantify their impact, time-event analysis was conducted to construct a Cox proportional hazards regression model to ensure the high success of fish passage. The model established a functional relationship between the risk of endpoint event occurrence and these influencing factors by using time-event analysis. To avoid multicollinearity among independent variables in the model, AIC was used to select the optimal model. The AIC is typically used for measuring the goodness of fit of statistical models. The analysis is usually terminated when ΔAIC is minimized. The Cox proportional hazards regression model, AIC criterion, and model weight calculate on formula used were as follows:

h(t,x) = h_0_(t) exp(βx^T^ + b*_i_*) (6)

AIC = 2k − 2ln(L)(7)

(8) $$wi=EXP(-0.5\Delta iAIC)/\sum_{i=1}^{n}\left(EXP(-0.5\Delta iAIC\right)\;$$

In the equation, h_0_(t) represents the baseline hazard rate of h(t,x) when vector x = 0. It is a quantity that can be estimated from the sample data, where x represents the influencing factors, b*_i_* the random effects, L the likelihood function, k the number of parameters, and w*_i_* the weights of the optimal model.

Data were processed using Microsoft Office, and Origin 2021 was used for academic data visualization. Data were analyzed using SPSS 19.0 and Rstudio 4.1.3. Differences among various datasets were assessed through one-way analysis of variance and nonparametric tests; *p* < 0.05 was considered to indicate statistical significance. Survival analysis using the Cox proportional hazards regression model was conducted in R, and the impact of various factors on fish migration was evaluated. The optimal model for fish migration was identified using the Akaike information criterion (AIC). Then, key factors influencing the fish migration efficiency were determined. Forest plots and alignment diagrams were generated for trend analysis and probability forecasting. Data are presented as the mean ± standard deviation (mean ± SD).

## 3. Results

### 3.1. Fish Resources in the Downstream River

**Figure 3:**
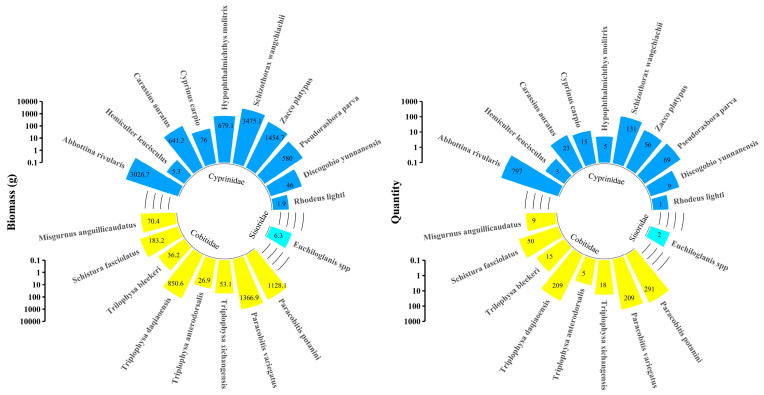
Fish resources in the river downstream of the dam. The figure shows the biomass (**left**) and abundance (**right**) of fish downstream of the Heishui River dam. Blue represents *Cyprinidae*, yellow represents *Cobitidae*, and green represents *Sisoridae*.

In total, 19 fish species were surveyed downstream of the Songxin Dam, including 10 from the *Cyprinidae* family, 8 from the *Cobitidae* family, and 1 from the *Balitoridae* family (Figure 3). The total biomass of *Cyprinidae* fish was 9986.0 g, *Cobitidae* fish accounted for 3715.4 g, and *Balitoridae* had only 6.3 g of biomass. In total, 1109, 806, and only 2 individuals of *Cyprinidae*, *Cobitidae*, and *Euchiloglanis* spp., respectively, were identified. *S. wangchiachii* accounted for 25.35% of the total biomass and 6.83% of the total quantity. Overall, the *Cyprinidae* fish species exhibited higher biomass and quantity than those of *Cobitidae* and *Balitoridae* fish, indicating that the downstream river environment below the dam is more suitable for *Cyprinidae* fish species.

### 3.2. Spatial and Temporal Patterns of Fish Passage over the Dam

**Figure 4:**
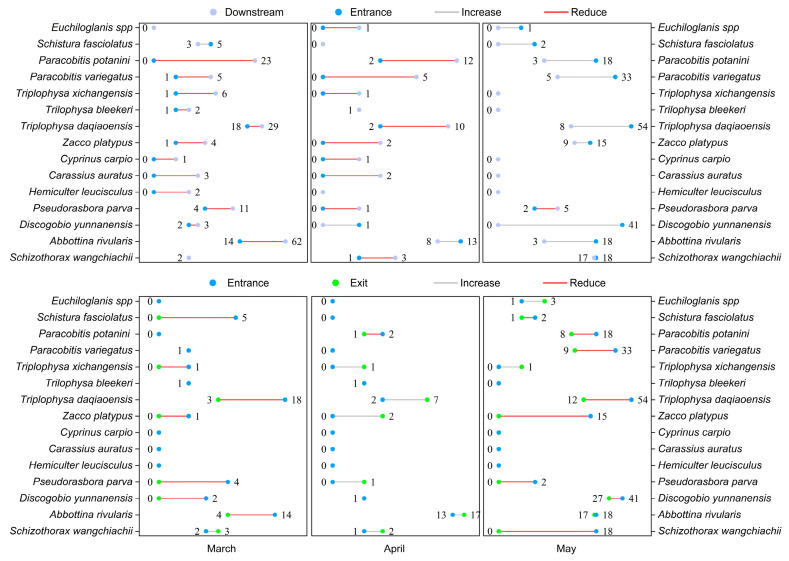
Spatio-temporal patterns of fish upstream passage at dams. The figure illustrates the spatiotemporal patterns of fish passage from the area near the dam to the fishway entrance and exit. Gray represents the downstream area, blue represents the entrance, and green represents the exit. The color of the lines connecting the points indicates whether the number of fish migrating upstream increases (gray) or decreases (red), The number represents the number of fish.

The spatiotemporal patterns of fish passage from the area near the dam to the fishway entrance and exit revealed differences in the attraction and passage efficiencies from March to May (Figure 4). From March to April, most fish were only attracted to the area near the dam, with very few finding their way to the fishway entrance, indicating that the attraction efficiency was low. In May, the number of fish at the fishway entrance significantly increased. This indicated that during this time, fish reached the area near the dam and located the fishway entrance for upstream migration. Regarding the passage efficiency within the fishway, the number and variety of fish passing through the fishway exit were higher in May than in March and April. In summary, the effectiveness of fish passage over the dam was higher in May than in March and April. Of note, 19 fish species were noted in the downstream river, and only 15 species could reach the area near the dam. Of the 15 species, only 12 could successfully reach the fishway entrance and exit. The number and variety of fish decreased throughout the upstream passage. This indicates that all fish species could not reach the fishway entrance and successfully pass over the dam.

### 3.3. Assessment of Fishway Entrance Attraction Efficiency

The fish release experiments in the downstream river section helped determine the impact of release locations on fish migration (Table 1). The average attraction rate was 15.7% at the fishway inlet. The attraction rate and attraction time at 1 m were 40% and 40.1 h, respectively, whereas at 30 m, they were 2.13% and 269.2 h, respectively. Furthermore, fish exhibited the highest speed at 10 m and the lowest speed at 30 m. Thus, as fish get closer to the fishway inlet, the chances of perceiving the inlet attraction flow and being attracted to it possibly increase, resulting in better upstream migration. Fish released farther away may not perceive the inlet attraction flow immediately or may need to continue upstream for some distance before they are attracted and migrate along the main channel.

### 3.4. Assessment of Fishway Passage Efficiency

**Figure 5:**
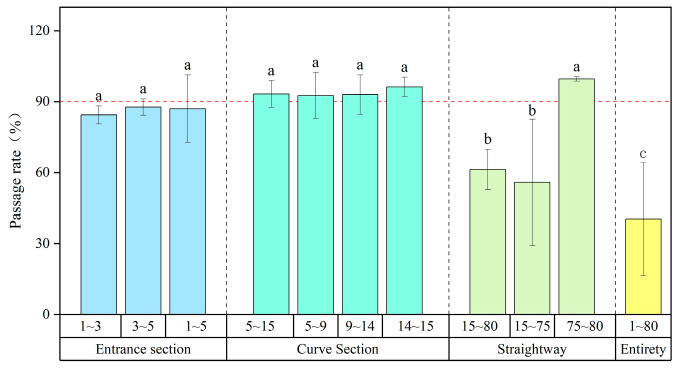
Passage rate in fishway pools. Blue represents the entrance section, green represents the curve section, cyan represents the straightway, and yellow represents the entirety. Different letters associated with the bars indicate significant differences (*p* < 0.05).

We here divided the vertical-slot fishway into three sections based on its structure and hydrodynamic differences: the entrance section (Pools 1–5), the curve section (Pools 5–15), and the straight section (Pools 15–80). Fish passage rates for each section were monitored and statistically analyzed through fish release experiments and RFID (Figure 5).

The entire fishway passage rate averaged 40.42%, which indicated that 40.42% of individuals that reached the fishway entrance could pass through it. Specifically, the curve section displayed the highest passage rates, exceeding 90% and thus indicating excellent passage effectiveness. The entrance section had a passage rate of 84.4%, slightly lower than that of the curve section. By contrast, the straight section exhibited a significantly lower passage rate of 61.32% than the other pool segments (*p* < 0.05). Thus, the curve section had the highest passage rate, followed by the entrance section and the straight section. However, the lower passage rate in the straight section does not necessarily indicate the presence of flow velocity barriers that prevent fish from migrating upstream.

**Figure 6:**
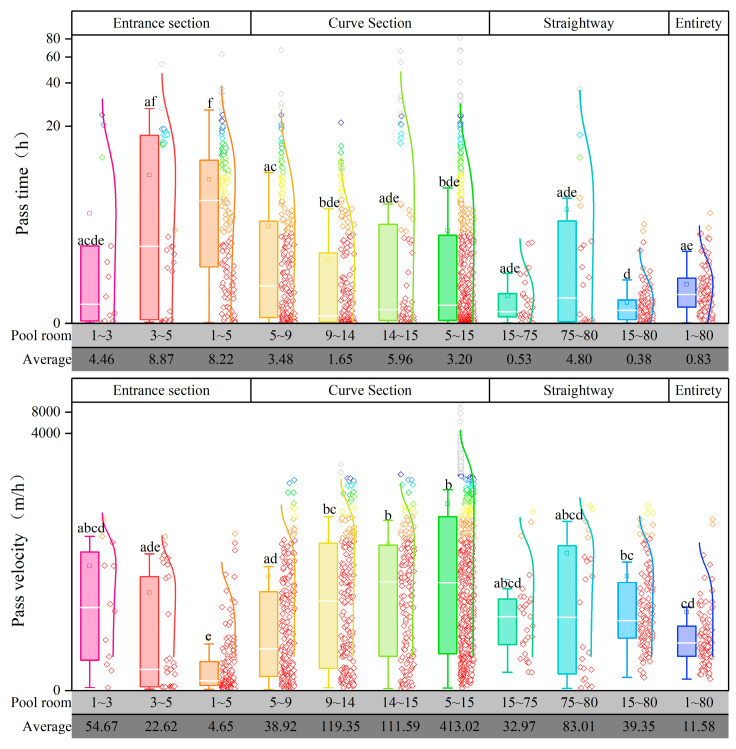
Passage time and velocity in fishway pools. The fishway was divided into several sections: the entrance section (1–5), the turning section (5–15), and the straight section (15–80). Intensified monitoring was conducted in different sections of the fishway to diagnose issues in specific pools. The figure presents the fish passage time (**top**) and speed (**bottom**) in different pool sections of the fishway, summarized and displayed as average values. Different letters associated with the bars indicate significant differences (*p* < 0.05).

To further diagnose the problems associated with pool rooms in the fishway, the passage time and speed for each pool room segment were statistically analyzed (Figure 6). The overall average passage time in the fishway was 0.83 h, with the passage time for the entrance, turning, and straight sections being 3.20, 0.83, and 0.34 h, respectively. The average passage speed in the entire fishway was 11.59 m/h, while that in the entrance, turning, and straight sections were 4.65, 413, and 39.35 m/h, respectively. Pool rooms 3–5 exhibited the lowest passage speed and the longest time. This indicated that obstacles were present in the entrance section of the fishway that made it difficult for the fish to swim upstream.

In summary, the straight section had a relatively low passage rate. However, fish in this section exhibited higher upstream speeds and required less time. By contrast, the entrance section exhibited a higher passage rate but longer upstream times and lower speeds. The curve section, on the other hand, exhibited a high passage rate, along with good upstream speed and timing. This trend suggests that obstacles in the entrance section may cause the fish to expend more energy during the upstream journey. When fish pass through the turning section and enter the straight section for upstream migration, although the straight section itself is suitable for fish migration, the fish have already expended energy and cannot maintain a consistently high migration efficiency, which ultimately leads to a decrease in the overall passage rate.

### 3.5. Diurnal and Nocturnal Rhythms in Fish Passage over the Weir

**Figure 7:**
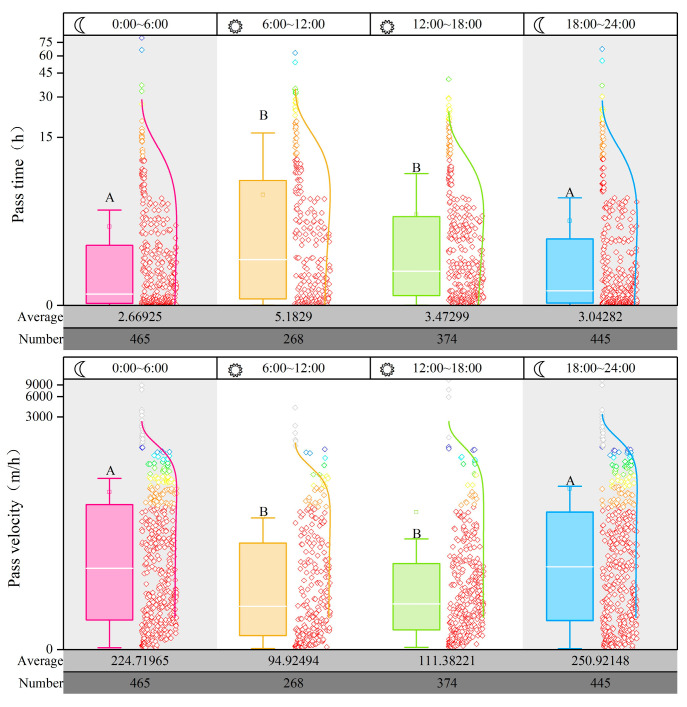
Circadian rhythms of fish upstream migration. The gray area represents nighttime, while the white area represents daytime. The figure displays the fish passage time (**top**) and speed (**bottom**) during different time periods. The average passage time, speed, and the number of successful passages for each time period are shown at the bottom of each graph. Different letters associated with the bars indicate significant differences (*p* < 0.05).

Diurnal and nocturnal rhythms of fish passage are crucial indicators for assessing and managing fishway effectiveness. Analysis of the upstream patterns and passage efficiency of fish during daytime and nighttime can offer a theoretical basis for fishway operation conditions and ecological scheduling. Here, we examined the diurnal and nocturnal rhythms of the passage of fish within the fishway by dividing the monitoring period into four time intervals: nighttime (18:00–24:00), early morning (0:00–6:00), morning (6:00–12:00), and afternoon (12:00–18:00). The average passage time, speed, and the number of successful passages for fish during each time interval were calculated (Figure 7).

Passage times were significantly longer during the daytime than during the nighttime (*p* < 0.05). Conversely, the passage speed was significantly lower during the daytime than during the nighttime (*p* < 0.05). Specifically, during the morning, fish had an average passage time and a speed of 5.18 h and 94.92 m/h, respectively. In the early morning, the average passage time and speed were 2.67 h and 224.72 m/h, respectively. Fish passing during the nighttime required half the time compared with that in the morning. Additionally, 465 and 445 successful passages occurred during the nighttime and early morning, respectively, whereas 268 and 374 successful passages occurred during the morning and afternoon, respectively. This indicates that fish are more active and motivated to migrate during the nighttime, and thus, they exhibit a preference for nocturnal migration.

### 3.6. Fish Upstream Behavior at Different Flow Rates and Water Temperatures

**Figure 8:**
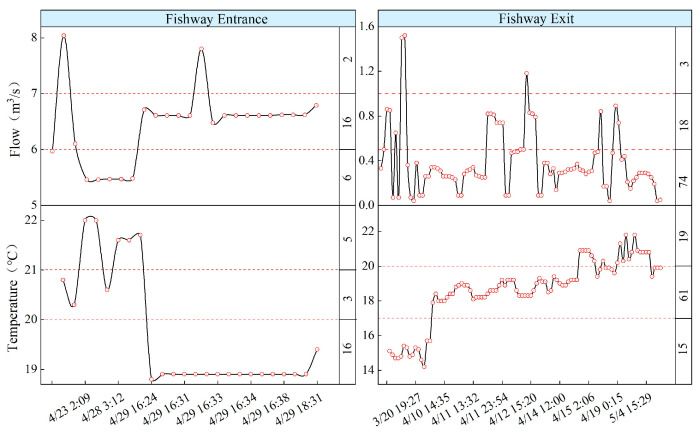
Fish upstream migration in response to flow and water temperature changes. The numbers in the right-hand box of the figure represent the count of successful fish passages within respective environmental factor intervals.

The upstream behavior of S. wangchiachii was monitored in response to environmental changes, thereby identifying the threshold values of environmental factors suitable for fish migration (Figure 8). The flow rate at the fishway entrance comprised both the water supplied through the channel and the fishway flow. Regarding the impact of flow rate and temperature on the number of fish attracted to the entrance, 24 short-finned eels were attracted to the fishway entrance. The number of fish that successfully reached the entrance was the highest and the attraction effect was the best when the total flow rate was between 6 and 7 m^3^/s and the temperature was between 19 °C and 20 °C. The best upstream passage effect was observed when the fishway flow rate was between 0 and 0.5 m^3^/s and the temperature was between 17 °C and 20 °C.

To identify key factors affecting fish attraction at the fishway entrance and fish passage effectiveness, factors related to the entrance attraction were considered from both biological and non-biological perspectives. These factors included fishway flow rate (FQ), supplementary water channel flow rate (BQ), temperature (T), fish body length (BL), the release location of fish downstream of the dam (Place), and diurnal/nocturnal rhythms (DR). Simultaneously, factors affecting the fishway passage efficiency such as FQ, fishway water level (WL), T, BL, and DR were considered. To identify key factors affecting fishway entrance attraction and passage efficiency, a survival analysis Cox proportional hazards regression model was constructed. The AIC was used to select the best-fitting model, thereby supporting the identification of critical factors influencing fishway entrance attraction and passage efficiency.

**Figure 9:**
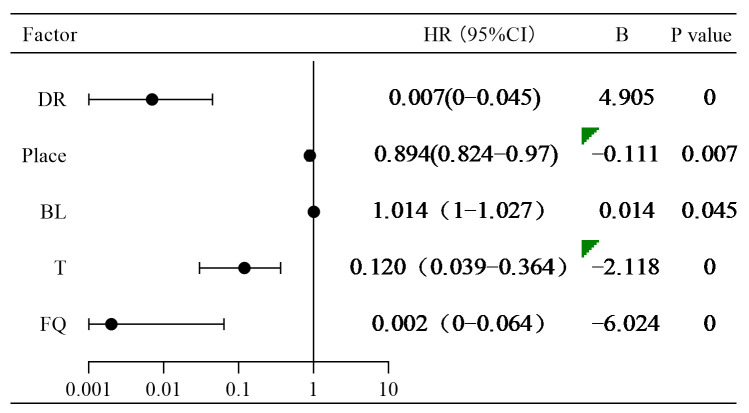
Forest diagram of fishway entrance attraction effect. HR represents hazard ratio, CI stands for confidence interval, and B represents regression coefficient; the same applies below.

Table 2 presents the optimal model for fishway entrance attraction effects. The model using the fishway flow rate, temperature, fish body length, release location, and diurnal/nocturnal rhythms as independent variables exhibited the lowest AIC value, indicating that it is the best-fitting model. According to the coefficient estimates in the Cox proportional hazards regression model (Figure 9), diurnal/nocturnal rhythms, release location, temperature, and fishway flow rate were inhibitory factors that negatively correlated with successful upstream passage. By contrast, fish body length was positively correlated with successful upstream passage. Specifically, the fishway entrance attraction rate was higher during the night than during the day (HR = 0.007, *p* < 0.01). When the release location was closer to the fishway entrance, the attraction effect at the fishway entrance was stronger (HR = 0.895, *p* < 0.01). Within a certain range, lower temperatures resulted in better attraction effects at the fishway entrance (HR = 0.120, *p* < 0.01). Similarly, lower fishway flow rates resulted in better attraction effects at the fishway entrance (HR = 0.002, *p* < 0.01). Fish body length was identified as a promoting factor, and within a specific range, larger fish exhibited better attraction effects at the fishway entrance (HR = 1.014, *p* < 0.05).

**Figure 10:**
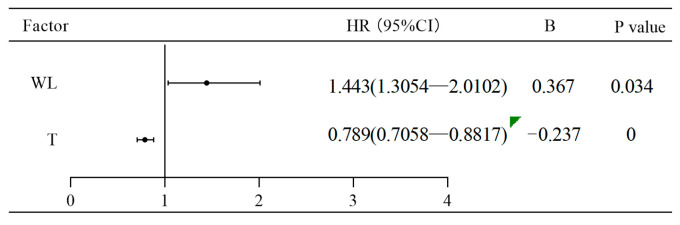
Forest diagram of fishway upstream passage effectiveness.

Table 3 presents the optimal model for fishway passage effectiveness. The model using temperature and water level as independent variables exhibited the lowest AIC value, confirming it as the optimal model. According to the coefficient estimates from the Cox hazard ratio regression model (Figure 10), temperature is an inhibitory factor and is negatively correlated with the probability of successful upstream migration. Within a certain range, lower temperatures led to better effectiveness of fishway passage (HR = 0.749, *p* < 0.001). Water level, on the other hand, was a promoting factor, and within specific limits, higher water levels resulted in better passage effectiveness (HR = 1.443, *p* = 0.034).

**Figure 11:**
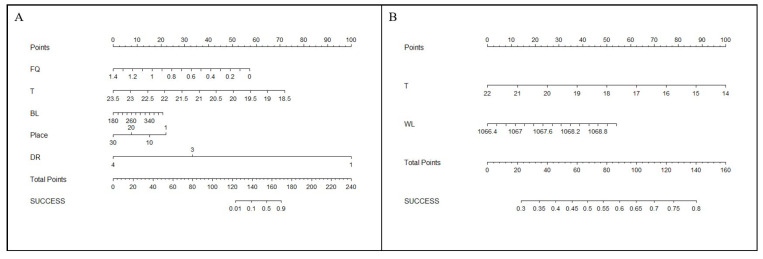
Column line graph for predicting fish upstream success probability. (**A**) Fishway entrance attraction success rate. (**B**) Fishway upstream passage success rate.

In this study, predictive probability contour plots for fishway entrance attraction and upstream success were established based on AIC values and the Cox hazard ratio regression model (Figure 11). These plots predict success probability in a scoring format by considering the influence of various factors. In the first column of the figure (Points), scores are assigned to each environmental factor. Summation of the scores for all factors provided the corresponding total score (Total Points). Finally, the success probability was estimated through this scoring system.

To validate the accuracy of these predictions, the example of a fishway entrance attraction was considered in this study. The total score for the relevant factors in this case was 145, which resulted in a predicted probability of 16%. Comparatively, the monitoring results indicated an average entrance attraction rate of 15.7%. The model’s predictions were closely aligned with the experimental conclusions, verifying the effectiveness of this method in predicting the success probability of fish upstream migration. This study can thus serve as a theoretical foundation for regulating fishway entrance attraction and passage efficiency, as well as for optimizing fishway designs.

## 4. Discussion

This study comprehensively analyzed and discussed key factors related to the fish passage efficiency and attraction mechanisms in vertical-slot fishways within the Heshuihe River ecosystem. This study offers valuable insights into various critical aspects of fish passage through the fishway and factors influencing this passage. These factors include downstream fish resources, spatiotemporal patterns of fish migration, fishway entrance attraction efficiency, passage efficiency, and other crucial factors influencing these processes.

### 4.1. Spatial and Temporal Patterns of Fish Migration

Riverine habitat ecosystems are a crucial player in sustaining fish survival and promoting fish community diversity [29]. Sampling near dams downstream can reveal the types of local fish populations and species that might use fishways [30]. By assessing fish resources in the downstream area of the Heishui River’s Songxin Dam, a diverse community comprising 19 fish species, with dominant representation from the *Cyprinidae*, *Cobitidae*, and *Gobionidae* families was identified. Among these, the *Cyprinidae* family was dominant in both biomass and quantity. These findings underscore the ubiquitous nature of *Cyprinidae* fish in habitats downstream of the dam. It thus indicates their frequent aggregation in the areas near the dam. This aggregation possibly occurs due to alterations in the fish habitat caused by dam water releases, ultimately resulting in differences in ecological habits among fish species [31].

Fish migratory motivations influence the effectiveness of fish passage through fishways. This effectiveness often exhibits strong seasonal patterns, with significant variations observed in the number of fish passing the fishway and the rate of successful fish passage during different time periods. Fish migration periods typically exhibit the highest upstream motivation and success rates [32,33]. Fish migration patterns analyzed over time and space highlighted intriguing variations in fish behavior. During the study period, fishway attraction and passage effectiveness in May surpassed those in March and April. This suggests that May is a favorable period for fish to seek and migrate to fishway entrances, and this seasonal difference may be observed because of the alignment of biological needs during fish migration with environmental conditions [34]. May is a potential period for reproduction and hatching, and fish exhibit a heightened migratory motivation in this month, which allows successful offspring propagation. Thus, increased attraction and passage effectiveness are observed at fishway entrances during this period [35].

Furthermore, engineers and scholars have exhibited increasing interest in improving fishways to accommodate a greater variety of species [36,37]. We found that during fish passage over the dam, the number of fish and their species gradually decreased from the river to the fishway entrance and then to the fishway exit. This indicates that not all fish can reach the fishway entrance and successfully pass over the dam. It highlights the physiological and behavioral challenges faced by fish during migration and the critical role of fishway design and flow management, which collectively influence the rate of successful fish passage [38]. These observations reveal the intricate strategies of fish migration behavior and provide substantial guidance for optimizing fishway design and management.

### 4.2. Fishway Efficiency Assessment and Diagnosis

During upstream migration, fishway entrance effectiveness and passage efficiency are two crucial challenges [14]. Notably, in many ecological populations and migration scenarios, the passage efficiency often exceeds the attraction efficiency [10]. The study results revealed that the average attraction rate at fishway entrances was approximately 15.7%, whereas the fish passage rate was 40.42%. This suggests that, for most fish, locating and successfully entering the fishway is more challenging than actually passing through the fishway. Moreover, significant differences in attraction rates were observed at various release points, primarily because of differences in how fish perceive and respond to the attraction flow at the entrance. However, fish have limited perception ranges for flow velocity [39]. Thus, when they are near the fishway entrance, the likelihood of perceiving the attraction flow and accurately locating the entrance for them is higher. Conversely, if fish are farther from the entrance, they may be unable to perceive the attraction flow or may need to continue migrating for some time before being attracted to enter and successfully traverse the fishway. Thus, the ability of fish to accurately locate the fishway entrance is pivotal in their successful upstream migration. In engineering practices, various methods such as hydraulic signals, sound, light, and bubble curtains are used to assist fish in precisely locating fishway entrances, thereby improving the attraction efficiency [40,41,42].

Monitoring the fishway passage efficiency is indispensable in addressing pool issues, optimizing fishway design, and enhancing the passage efficiency [43]. When fish behavior within the fishway is analyzed in detail, problematic pools can be accurately identified and corresponding solutions can be developed. This would optimize the fishway structure and improve the passage efficiency. The vertical-slot fishway was divided into the entrance, turning, and straight sections based on the fishway structure and hydraulic differences.

The entrance section exhibited a higher passage rate but longer passage time and slower speed, whereas the straight and turning sections demonstrated better passage speed and time, albeit with a relatively lower passage rate. This indicates that fish face challenges during upstream migration to the entrance section, where continuous counterflow increases energy consumption [44], weakening the fish’s ability to migrate within the fishway. The issues in the entrance section may be attributable to water-level differences, as the water level differential between upstream and downstream is among the key driving factors affecting fishway hydraulic characteristics [45]. When the downstream of the Songxin Hydropower Station was used as an example, the lower river water level led to a significantly lower water level in the entrance section than in the straight and turning sections. However, this reduced water level creates high-velocity obstacle zones, affecting fish’s upstream migration ability.

Furthermore, water level was identified as a facilitating factor by using the Cox hazard ratio regression model. This indicated that, within a specific range, higher water levels result in better fishway passage efficiency. This conclusion further validates the prominence of increasing water levels. Engineering solutions may include implementing water supplementation at the fishway entrance, which can extend the attraction range of the entrance section to enhance the passage efficiency [46] and elevate water levels within the fishway, thereby reducing the impact of high-velocity obstacle zones, mitigating fish energy consumption, and thus improving the overall fishway passage efficiency.

### 4.3. Identification of Key Factors Affecting Fish Upstream Migration

Biotic and abiotic factors play essential roles in fish’s growth activities, development, behavior, and physiology [47,48]. Among various fish behavior-affecting environmental factors, water flow is crucial and is a fundamental environmental condition for fish survival [49]. Fish behavior and swimming patterns vary according to changes in water flow [24]. The study results indicate that fishway flow is negatively correlated to the attraction effect at the fishway entrance within a certain range. Under high-flow conditions, the dynamic performance of water flow provides clear guiding signals for fish, which allow them to find the fishway entrance more quickly. However, in excessively high-flow conditions, fish may experience significant hydrodynamic impacts, which leads to increased energy consumption and adversely affects the upstream efficiency [50]. Therefore, flow must be controlled within a reasonable range to achieve a balance between guiding effectiveness and passage capacity. On analyzing the relationship between the number of successfully passed fish during the upstream process and flow, we noted that the optimal attraction effect at the fishway entrance was achieved when the total entrance flow was within 6–7 m^3^/s, while the best passage efficiency in the fishway was achieved when the flow is within 0–0.5 m^3^/s. This conclusion provides valuable scientific evidence for fishway operational management, river connectivity restoration, and ecological scheduling measures.

Migration of adult fish upstream for spawning and of juvenile fish downstream for feeding are crucial strategies in the fish migration life cycle, which are closely related to their ecological adaptation and survival needs [51,52]. During monitoring, differences in species and fish size were observed in the upstream migration of fish. Specifically, the size of fish captured in the river areas adjacent to the fishway gradually increased, with the most significant size difference observed at the fishway entrance. This may be attributable to the increase in water flow as fish approach the fishway, which affects the swimming performance of smaller individuals [53,54]. Larger fish have a higher success rate in upstream migration than smaller fish [36,55]. The results emphasize that body length is also a key facilitating factor for the attraction effect at the fishway entrance. Within a specific range, a larger body size significantly enhances the attraction effect at the fishway entrance. Larger fish may be more inclined to be sexually mature and have more motivation to migrate upstream [7]. However, body length is not a key factor affecting fishway passage effectiveness. This is because fish have a weaker swimming ability when they enter the fishway entrance, which makes the differences in body size among fish passing through the fishway less significant.

Temperature is a guiding factor prompting fish metabolic systems to respond appropriately [56]. Changes in temperature can directly affect the physiological state and behavioral patterns of fish, which further influence their perception and response to the fishway [57]. Differences in the upstream behavior of fish were observed at different water temperatures. Within a certain range, both the attraction effect at the entrance and the passage efficiency increased with a decrease in temperature. This temperature effect may be closely related to the physiological and environmental adaptability of fish. Within a suitable temperature range, fish can better maintain their metabolic balance, neural activity, and muscle coordination, which results in more positive behavioral patterns. However, when the temperature exceeds this range, physiological processes may be disrupted, thereby restricting fish behavior [58]. There is a critical point, the optimal temperature, at which fish perform best. The study results suggest that the optimal water temperature for the attraction effect at the fishway entrance and the passage efficiency was 19-20 °C. Beyond this range, fish may experience a reduction in appetite, activity, and ability to locate the fishway entrance because of physiological stress, thereby reducing the passage efficiency [59,60].

Diurnal and nocturnal rhythms are key environmental factors influencing the upstream efficiency of fish passage [61]. During upstream migration, fish exhibit diurnal and nocturnal variations in their behavior [62]. In this study, the number of fish passing was lower during the daytime than during the nighttime. Specifically, in the morning and afternoon, which are daytime periods, fish took longer time to pass, and their upstream speed was also slower. This suggests that during the daytime, fish tend to linger within the fishway to adapt to environmental changes, find better passage routes, or engage in other activities. However, during the early morning and nighttime, which are nighttime periods, fish took less time to pass, but their upstream speed significantly increased. This may be because, at night, fish are more actively seeking and entering the fishway to cope with water flow and other environmental influences. This is consistent with earlier observations in fish migration behavior. Fish are more active at night, which may be a response to avoid predators, reduce hydrodynamic pressure, or follow natural migratory rhythms [63,64]. These findings regarding fish diurnal and nocturnal rhythms have substantial implications for assessing and managing fishway effectiveness. First, according to the observed patterns of fish upstream movement during different times of the day, fishway operation may be scheduled more effectively. For example, releasing passage flow during the night may help meet the upstream migration needs of fish more effectively. Additionally, for fishway maintenance and management, more frequent monitoring and repairs must be conducted during the nighttime when fish are more active to ensure its functionality [65].

This study focused on the vertical-slot fishway in the Heshuihe River. We conducted a comprehensive analysis of the fish passage efficiency and its influencing factors, covering the entire stretch from the river downstream of the dam to the fishway entrance and exit. The mechanisms underlying the impact of fish migration behavior, flow velocity, water temperature, diurnal and nocturnal rhythms, and other environmental factors on fishway effectiveness were evaluated. Differences between the fishway entrance attraction and the passage efficiency, along with the ecological reasons for these disparities, were unveiled. By constructing a Cox hazard ratio model, key factors affecting the fish upstream efficiency were identified, based on which we established a probability prediction line chart, thereby providing substantial guidance for fishway management and optimization. In engineering practice, this study’s findings are expected to inform fishway design and flow management to support successful fish migration and the sustainable development of ecosystems.

## 5. Conclusions

This study monitored 19 fish species downstream of the Songxin Dam, revealing that the region’s environmental conditions are more favorable for Cyprinidae, which exhibited the highest biomass and abundance. The research indicated that May is the optimal period for fish migration, with nighttime migration success rates exceeding those during the day. Fish passage success rates decreased progressively from the fishway entrance to the exit, with only 12 species successfully completing the migration. The vertical-slot fishway demonstrated varying passage efficiencies across its sections, with the curve section achieving the highest success rates, followed by the entrance and straight sections. Despite the lower passage rate in the straight section, fish exhibited the fastest upstream speed there, indicating the absence of significant barriers.

A multifactorial Cox regression model identified key factors influencing fishway performance, showing that proximity to the fishway entrance, lower temperatures, reduced flow rates, and larger fish body length increased attraction rates, while higher water levels contributed to improved passage efficiency. Based on these factors, a nomogram was developed to accurately predict the success probability of fishway attraction and passage, providing valuable insights into optimizing fishway design and operation.

## Figures and Tables

**Table 1:** Assessment of fishway entrance attraction effect.

Distance (m)	Attraction Rate (%)	Attraction Time (h)	Attraction Speed (m/h)
1	40.00	40.1 ± 1.1	0.04 ± 0.05
10	6.67	80.29 ± 3.9	0.49 ± 0.29
20	14.00	194.3 ± 4.32	0.16 ± 0.15
30	2.13	269.2	0.11

**Table 2:** Optimal model selection of fishway entrance attraction effect based on AIC criterion.

Number	Model	AIC	△_I_AIC	w_i_	w_i_/w_j_
1	FQ + BQ + T + BL + Place + DR	123.21	1.97	0.271901	2.677812
**2**	**FQ + T + BL + Place + DR**	**121.24**	**0**	**0.728099**	

**Table 3:** Optimal model selection of fishway passing efficiency based on AIC criterion.

Number	Model	AIC	△_I_AIC	w_i_	w_i_/w_j_
1	FQ + T + BL + DR + WL	584.11	1.98	0.13510908	2.691234472
2	FQ + T + DR + WL	582.13	1.22	0.197567926	1.840431399
3	T + DR + WL	580.91	0.36	0.30371278	1.197217363
4	T + WL	580.55	0	0.363610214	

## Data Availability

Some or all data, models, or codes that support the findings of this study are available from the corresponding author upon reasonable request.

## References

[1] Doehring K., Young R.G., McIntosh A.R. (2011). Factors affecting juvenile galaxiid fish passage at culverts. Mar. Freshw. Res..

[2] Hall C.J., Jordaan A., Frisk M.G. (2011). The historic influence of dams on diadromous fish habitat with a focus on river herring and hydrologic longitudinal connectivity. Landsc. Ecol..

[3] García A., Jorde K., Habit E., Caamaño D., Parra O. (2011). Downstream environmental effects of dam operations: Changes in habitat quality for native fish species. River Res. Appl..

[4] Newbold L.R., Shi X., Hou Y., Han D., Kemp P.S. (2016). Swimming performance and behaviour of bighead carp (*Hypophthalmichthys nobilis*): Application to fish passage and exclusion criteria. Ecol. Eng..

[5] Santos J.M., Ferreira M.T., Pinheiro A.N., Bochechas J.H. (2006). Effects of small hydropower plants on fish assemblages in medium-sized streams in central and northern Portugal. Aquat. Conserv. Mar. Freshw. Ecosyst..

[6] Dodd J.R., Cowx I.G., Bolland J.D. (2017). Efficiency of a nature-like bypass channel for restoring longitudinal connectivity for a river-resident population of brown trout. J. Environ. Manag..

[7] Pennock C.A., Bender D., Hofmeier J., Mounts J.A., Waters R., Weaver V.D., Gido K.B. (2018). Can fishways mitigate fragmentation effects on Great Plains fish communities?. Can. J. Fish. Aquat. Sci..

[8] Tummers J.S., Hudson S., Lucas M.C. (2016). Evaluating the effectiveness of restoring longitudinal connectivity for stream fish communities: Towards a more holistic approach. Sci. Total Environ..

[9] Qin Y., Wei Q., Ji Q., Li K., Liang R., Wang Y. (2023). Determining the position of a fish passage facility entrance based on endemic fish swimming abilities and flow field. Environ. Sci. Pollut. Res..

[10] Hershey H. (2021). Updating the consensus on fishway efficiency: A meta-analysis. Fish Fish..

[11] Noonan M.J., Grant J.W.A., Jackson C.D. (2012). A quantitative assessment of fish passage efficiency. Fish Fish..

[12] Quaranta E., Katopodis C., Comoglio C. (2019). Effects of bed slope on the flow field of vertical slot fishways. River Res. Appl..

[13] Chen A., Wu M., Chen K.-q., Sun Z.-y., Shen C., Wang P.-y. (2016). Main issues in research and practice of environmental protection for water conservancy and hydropower projects in China. Water Sci. Eng..

[14] Cooke S.J., Hinch S.G. (2013). Improving the reliability of fishway attraction and passage efficiency estimates to inform fishway engineering, science, and practice. Ecol. Eng..

[15] Quintella B., Mateus C.S., Alexandre C.M., Cardoso G.R., Belo A.F., Pereira E.D., Almeida P.R. Session c7: Passage efficiency and behavior of adult sea lamprey in a vertical-slot fishway. Proceedings of the International Conference on Engineering and Ecohydrology for Fish Passage 2015.

[16] Garavelli L., Linley T.J., Bellgraph B.J., Rhode B.M., Janak J.M., Colotelo A.H. (2019). Evaluation of passage and sorting of adult Pacific salmonids through a novel fish passage technology. Fish. Res..

[17] LeRoy J.Z., Davis J.J., Shanks M.R., Jackson P.R., Murphy E.A., Baxter C.L., McInerney M.K. (2019). Efficacy of increasing discharge to reduce tow-mediated fish passage across an electric dispersal barrier system in a confined channel. J. Great Lakes Res..

[18] Baumgartner L.J., Barlow C., Mallen-Cooper M., Boys C., Marsden T., Thorncraft G., Roy M. (2021). Achieving fish passage outcomes at irrigation infrastructure; a case study from the Lower Mekong Basin. Aquac. Fish..

[19] Tan J., Tan H., Goerig E., Ke S., Huang H., Liu Z., Shi X. (2021). Optimization of fishway attraction flow based on endemic fish swimming performance and hydraulics. Ecol. Eng..

[20] Chen M., An R., Li J., Li K., Li F. (2019). Identifying operation scenarios to optimize attraction flow near fishway entrances for endemic fishes on the Tibetan Plateau of China to match their swimming characteristics: A case study. Sci. Total Environ..

[21] Fritts A.K., Knights B.C., Stanton J.C., Milde A.S., Vallazza J.M., Brey M.K., Lamer J.T. (2021). Lock operations influence upstream passages of invasive and native fishes at a Mississippi River high-head dam. Biol. Invas..

[22] Benitez J.P., Ovidio M. (2018). The influence of environmental factors on the upstream movements of rheophilic cyprinids according to their position in a river basin. Ecol. Freshw. Fish..

[23] Goerig E., Wasserman B.A., Castro-Santos T., Palkovacs E.P. (2020). Body shape is related to the attempt rate and passage success of brook trout at in-stream barriers. J. Appl. Ecol..

[24] Harbicht A.B., Castro-Santos T., Gorsky D., Hand D.M., Fraser D.J., Ardren W.R. (2018). Environmental, anthropogenic, and dietary influences on fine-scale movement patterns of Atlantic salmon through challenging waters. Can. J. Fish. Aquat. Sci..

[25] Goerig E., Castro-Santos T. (2017). Is motivation important to brook trout passage through culverts?. Can. J. Fish. Aquat. Sci..

[26] Wang Y., Wai O.W.H., Chen Q. (2021). Laboratory study on fish behavioral response to meandering flow and riffle-pool sequence driven by deflectors in straight concrete flood channels. J. Hydrol..

[27] Bayse S.M., McCormick S.D., Castro-Santos T. (2019). How lipid content and temperature affect American shad (*Alosa sapidissima*) attempt rate and sprint swimming: Implications for overcoming migration barriers. Can. J. Fish. Aquat. Sci..

[28] Ding R.H. (1994). The Fishes of Sichuan.

[29] McCain J.S.P., Rangeley R.W., Schneider D.C., Lotze H.K. (2016). Historical abundance of juvenile commercial fish in coastal habitats: Implications for fish habitat management in Canada. Mar. Policy.

[30] Aparicio E., Pintor C., Duran M.C., Carmona Catot G. (2012). Fish passage assessment at the most downstream barrier of the Ebro River (NE Iberian Peninsula). Limnetica.

[31] Smith S.C.F., Meiners S.J., Hastings R.P., Thomas T., Colombo R.E. (2017). Low-head dam impacts on habitat and the functional composition of fish communities. River Res. Appl..

[32] Keefer M.L., Peery C.A., Bjornn T.C., Jepson M.A., Stuehrenberg L.C. (2004). Hydrosystem, dam, and reservoir passage rates of adult Chinook salmon and steelhead in the Columbia and Snake rivers. Trans. Am. Fish. Soc..

[33] Yoon J.D., Kim J.H., Yoon J., Baek S.H., Jang M.H. (2015). Efficiency of a modified Ice Harbor-type fishway for Korean freshwater fishes passing a weir in South Korea. Aquat. Ecol..

[34] Epple T., Friedmann A., Wetzel K.-F., Born O., Müller G. (2022). The migration of four salmonid species through fish bypass channels depending on environmental factors. Environ. Biol. Fish..

[35] Tamario C., Sunde J., Petersson E., Tibblin P., Forsman A. (2019). Ecological and evolutionary consequences of environmental change and management actions for migrating fish. Front. Ecol. Evol..

[36] Katopodis C., Williams J.G. (2012). The development of fish passage research in a historical context. Ecol. Eng..

[37] Sanz-Ronda F.J., Bravo-Córdoba F.J., Fuentes-Pérez J.F., Castro-Santos T. (2016). Ascent ability of brown trout, *Salmo trutta*, and two Iberian cyprinids− Iberian barbel, *Luciobarbus bocagei*, and northern straight-mouth nase, *Pseudochondrostoma duriense*− in a vertical slot fishway. Knowl. Manag. Aquat. Ecosyst..

[38] Lopes J.d.M., Alves C.B.M., Peressin A., Pompeu P.S. (2021). Dazed and confused: Behavioural constraints impose major challenges to fish passage in the neotropics. Aquat. Conserv. Mar. Freshwater Ecosyst..

[39] Xie O., Sun Z., Shen C. (2023). A study on flow field characteristics of a self-propelled robot fish approaching static obstacles based on artificial lateral line. Bioinspirat. Biomimet..

[40] Burnett N.J., Hinch S.G., Bett N.N., Braun D.C., Casselman M.T., Cooke S.J., Minke-Martin V. (2017). Reducing carryover effects on the migration and spawning success of sockeye salmon through a management experiment of dam flows. River Res. Appl..

[41] Green T.M., Lindmark E.M., Lundström T.S., Gustavsson L.H. (2011). Flow characterization of an attraction channel as entrance to fishways. River Res. Appl..

[42] Vowles A.S., Kemp P.S. (2012). Effects of light on the behaviour of brown trout (*Salmo trutta*) encountering accelerating flow: Application to downstream fish passage. Ecol. Eng..

[43] Bao J., Li W., Zhang C., Mi X., Li H., Zhao X., Duan M. (2019). Quantitative assessment of fish passage efficiency at a vertical-slot fishway on the Daduhe River in Southwest China. Ecol. Eng..

[44] Golpira A., Baki A.B.M., Zhu D.Z. (2020). Higher-order velocity moments, turbulence scales and energy dissipation rate around a boulder in a rock-ramp fish passage. Sustainability.

[45] Quaranta E., Katopodis C., Revelli R., Comoglio C. (2017). Turbulent flow field comparison and related suitability for fish passage of a standard and a simplified low-gradient vertical slot fishway. River Res. Appl..

[46] Schuetz C., Henning M., Czerny R., Herbst M., Pitsch M. (2021). Addition of auxiliary discharge into a fishway–a contribution to fishway design at barrages of large rivers. Ecol. Eng..

[47] de Oliveira Mesquita F., Godinho H.P., de Azevedo P.G., Young R.J. (2008). A preliminary study into the effectiveness of stroboscopic light as an aversive stimulus for fish. Appl. Anim. Behav. Sci..

[48] Vetter B.J., Murchy K.A., Cupp A.R., Amberg J.J., Gaikowski M.P., Mensinger A.F. (2017). Acoustic deterrence of bighead carp (*Hypophthalmichthys nobilis*) to a broadband sound stimulus. J. Great Lakes Res..

[49] Castro-Santos T. (2005). Optimal swim speeds for traversing velocity barriers: An analysis of volitional high-speed swimming behavior of migratory fishes. J. Exp. Biol..

[50] Ovidio M., Sonny D., Dierckx A., Watthez Q., Bourguignon S., de Le Court B., Benitez J.P. (2017). The use of behavioural metrics to evaluate fishway efficiency. River Res. Appl..

[51] Aarestrup K., Baktoft H., Thorstad E.B., Svendsen J.C., Höjesjö J., Koed A. (2015). Survival and progression rates of anadromous brown trout kelts *Salmo trutta* during downstream migration in freshwater and at sea. Mar. Ecol. Progr. Ser..

[52] Benitez J.-P., Dierckx A., Matondo B.N., Rollin X., Ovidio M. (2018). Movement behaviours of potamodromous fish within a large anthropised river after the reestablishment of the longitudinal connectivity. Fish. Res..

[53] Bunt C.M., Castro-Santos T., Haro A. (2012). Performance of fish passage structures at upstream barriers to migration. River Res. Appl..

[54] Silva A.T., Santos J.M., Ferreira M.T., Pinheiro A.N., Katopodis C. (2011). Effects of water velocity and turbulence on the behaviour of Iberian barbel (*Luciobarbus bocagei*, Steindachner 1864) in an experimental pool-type fishway. River Res. Appl..

[55] White L.J., Harris J.H., Keller R.J. (2011). Movement of three non-salmonid fish species through a low-gradient vertical-slot fishway. River Res. Appl..

[56] Liu Q., Zhang P., Cheng B., Li Y., Li J., Zhou H., Lu Y. (2021). Incorporating the life stages of fish into habitat assessment frameworks: A case study in the Baihetan Reservoir. J. Environ. Manag..

[57] Lehman B., Huff D.D., Hayes S.A., Lindley S.T. (2017). Relationships between Chinook salmon swimming performance and water quality in the San Joaquin River, California. Trans. Am. Fish. Soc..

[58] Samaras A., Papandroulakis N., Lika K., Pavlidis M. (2018). Water temperature modifies the acute stress response of European sea bass, *Dicentrarchus labrax* L. (1758). J. Therm. Biol..

[59] Cai L., Liu G., Taupier R., Fang M., Johnson D., Tu Z., Huang Y. (2014). Effect of temperature on swimming performance of juvenile Schizothorax prenanti. Fish Physiol. Biochem..

[60] Salinger D.H., Anderson J.J. (2006). Effects of water temperature and flow on adult salmon migration swim speed and delay. Trans. Am. Fish. Soc..

[61] Pereira E., Cardoso G.R., Quintella B.R., Mateus C.S., Alexandre C.M., Oliveira R.L., Batista C.M. (2019). Proposals for optimizing sea lamprey passage through a vertical-slot fishway. Ecohydrology.

[62] Baker N.J., Boubée J., Lokman P.M., Bolland J.D. (2020). Evaluating the impact of hydropower on downstream migrating anguillid eels: Catchment-wide and fine-scale approaches to identify cost-effective solutions. Sci. Total Environ..

[63] Hou Y., Yang Z., An R., Cai L., Chen X., Zhao X., Zou X. (2019). Water flow and substrate preferences of Schizothorax wangchiachii (Fang, 1936). Ecol. Eng..

[64] López-Olmeda J.F., López-García I., Sánchez-Muros M.J., Blanco-Vives B., Aparicio R., Sánchez-Vázquez F.J. (2012). Daily rhythms of digestive physiology, metabolism and behaviour in the European eel (*Anguilla anguilla*). Aquac. Int..

[65] Lagarde R., Teichert N., Boussarie G., Grondin H., Valade P. (2015). Upstream migration of amphidromous gobies of La Réunion Island: Implication for management. Fish. Manag. Ecol..

